# Retrospective Analysis of Balance Parameters in Pregnant Women: A Sub-Analysis of a Randomized Controlled Trial

**DOI:** 10.3390/jcm14061892

**Published:** 2025-03-11

**Authors:** Dilek Bayraktar, Seçkin Şenışık, Ayşe Kayalı Vatansever, Ömer Faruk Dadaş, Fuat Akercan

**Affiliations:** 1 Department of Orthopedics and Traumatology, Ege University, 35040 Bornova, Türkiye; dilekbayraktar8@gmail.com; 2 Department of Sports Medicine, Ege University, 35040 Bornova, Türkiye; seckinsnsk@gmail.com; 3 Department of Physiotherapy and Rehabilitation, Bakırçay University, 35665 Menemen, Türkiye; 4 Department of Biostatistics and Medical Informatics, Ege University, 35040 Bornova, Türkiye; omerfarukdadas@gmail.com; 5 Department of Obstetrics and Gynecology, Ege University, 35040 Bornova, Türkiye; fuat.akercan@ege.edu.tr

**Keywords:** pregnancy, exercise, physical activity

## Abstract

**Objectives**: Altered body biomechanics during pregnancy can lead to balance impairments and an increased risk of falls. Clinical exercise interventions can help regulate these biomechanical changes. **Methods**: A total of 101 pregnant participants (exercise group: *n* = 50; control group: *n* = 51) were retrospectively analyzed over an 8-week follow-up period. Single-leg balance parameters, including AP sway, ML sway, total body sway, OSD, and center of pressure velocity and acceleration, were assessed considering limb dominance. Measurements were taken at baseline and week 8. Repeated-measures ANOVA was used to analyze time–group interactions, with significance set at *p* < 0.001. The biomechanical impacts of participant height and body mass on center of pressure dynamics were also considered. **Results**: The exercise group (EG) demonstrated significant improvements in all balance parameters compared to the control group (CG) (*p* < 0.001), except for non-dominant anterior-posterior (AP) sway (*p* = 0.512). In the EG, medio-lateral (ML) and AP sway of the non-dominant limb were minimized, whereas these parameters were significantly increased in the CG. Although both groups exhibited an increased one-leg stance duration (OSD), the improvement was more pronounced in the EG. The controlled improvements observed in the EG suggest a protective effect of exercise on balance, particularly in the dominant limb. **Conclusions**: Clinical exercise interventions during pregnancy enhance balance parameters, reduce fall risk, and improve functional mobility. These findings suggest that structured exercise programs not only support maternal well-being but also improve reactive balance control. Given the biomechanical changes throughout pregnancy, future studies should examine the center of pressure velocity, acceleration, and the influence of maternal anthropometrics on postural stability to refine exercise recommendations.

## 1. Introduction

Pregnancy represents a dynamic physiological process involving continuous anatomical and functional adaptations. Throughout the 40-week gestational period, the maternal body undergoes complex modifications to accommodate a growing fetus. This process necessitates the consideration of numerous physiological, biomechanical, and clinical parameters [1]. The hormone relaxin, which remodels pelvic connective tissue and activates the collagenolytic system, begins to increase from the very first days of pregnancy and reaches its peak levels by the 12th week [2]. The pubic symphysis and sacroiliac joints undergo preparation for childbirth, with the pubic symphysis beginning to widen between the 10th and 12th weeks of pregnancy. As the pelvis and hip joints undergo significant structural changes, the movements of the trunk and lower extremities are also altered [3]. In addition to all these changes, fetal growth induces alterations in body posture [4]. These include the posterior tilt of the head, the anterior tilt of the pelvis, increased lumbar lordosis, knee hyperextension, and a reduction in foot arch height. These changes result in a shift in the center of balance [5].

During pregnancy, a stance with an increased distance between the feet is commonly adopted to enhance the base of support. This adjustment improves balance and reduces mediolateral sway [6]. In the second and third trimesters of pregnancy, step length decreases. During the third trimester, step width increases, along with prolonged durations of the stance phase and double support periods [7]. This gait adaptation is crucial for the safety of pregnant women. Notably, the incidence of fall-related problems experienced by pregnant women while walking is comparable to that observed in women over the age of 70 [8]. A study by Dunning et al. reported that pregnant individuals have a 28% risk of experiencing falls throughout the duration of pregnancy [9]. It has been reported that this rate may rise to 70% during the third trimester. Furthermore, the risk of falls in pregnant individuals is 2.3 times higher than that of non-pregnant individuals of the same age [10].

The findings indicate an increased demand for hip abductors and extensors, as well as ankle plantar flexors, during walking in pregnancy [11]. Huang et al., in their gait analysis study comparing pregnant and non-pregnant women, reported significant differences specifically in knee abduction angles and knee and hip internal rotation angles [12]. During the single support period, which is part of the swing phase constituting 40% of the gait cycle, stronger hip abductors are required [6,13]. Moreover, during functional activities, it is likely that the support of the body’s center of mass is more stable on the dominant side [14]. Our review of the literature revealed no studies specifically examining the impact of clinical exercise training on single-leg balance during pregnancy. However, existing evidence highlights the necessity of clinical exercise interventions to enhance the ability of pregnant individuals to cope with increased ground reaction forces during functional and static activities. It is suggested that maintaining adequate tone and strength of the pelvic and lower extremity muscles through such interventions can help prevent falls [15,16,17]. The aim of this study was to investigate the effects of a clinical exercise program implemented during pregnancy on single-leg stance duration, as well as on mediolateral and anteroposterior sway.

## 2. Materials and Methods

### 2.1. Participants

This study was conducted retrospectively by reanalyzing data from a previously conducted single-blind randomized controlled trial titled “The Effects of Clinical Exercise Training on Foot Plantar Pressure, Subtalar Joint, and Gait Cycle in Pregnant Women”, performed at the same center [18]. Ethical approval was obtained from the Institutional Ethics Committee (23-11.1T/42). The inclusion criteria were as follows: (i) aged between 18 and 40 years, (ii) absence of pregnancy-related complications, and (iii) gestational age between the 12th and 24th weeks. The exclusion criteria included the following: (i) a history of lower extremity, pelvic, or spinal surgery; (ii) chronic pain in the lower extremities, pelvis, or spine persisting for more than six months; and (iii) any diagnosis of fetal growth restriction. A total of 101 healthy pregnant participants were included in the study, with 50 assigned to the exercise group (EG) and 51 to the control group (CG).

The participants in the exercise group underwent supervised clinical exercise training for 8 weeks, consisting of 16 sessions conducted twice per week under the supervision of a specialist physiotherapist. Each 45 min session included a warm-up, a main exercise phase, and a cool-down, with the program tailored to be pregnancy-appropriate and individualized based on each participant’s gestational week [18]. The warm-up phase consisted of light walking and deep diaphragmatic breathing exercises to promote circulation, neuromuscular activation, and relaxation. The main exercise phase included core stabilization exercises, targeted exercises for the pelvic floor and hip muscles, and therapeutic exercises combined with breathing techniques to strengthen the lower extremity muscles. Strength training was performed using dumbbells and resistance bands, with intensity regulated according to the Modified Borg Scale (RPE 4–6) [18,19]. The core stabilization exercises incorporated large and small exercise balls to enhance trunk control and postural stability, while the pelvic and hip strengthening exercises focused on improving lumbopelvic stability. The balance and proprioception training involved single-leg stance exercises and controlled weight-shifting movements to address pregnancy-related postural adaptations. The session concluded with a 10 min cool-down period consisting of gentle stretching exercises targeting major muscle groups to aid recovery and reduce post-exercise muscle tension. All exercise sessions were conducted in accordance with the WHO’s health recommendations for pregnancy [17,18,19]. Unlike the control group, which was only advised to engage in at least 150 min of moderate-intensity aerobic activity per week (primarily walking), the exercise group followed a supervised, structured, and targeted exercise intervention addressing pregnancy-specific biomechanical changes. The details of the exercise protocol can be found in the Supplementary Materials (File S1).

### 2.2. Data Collection

**Figure 1:**
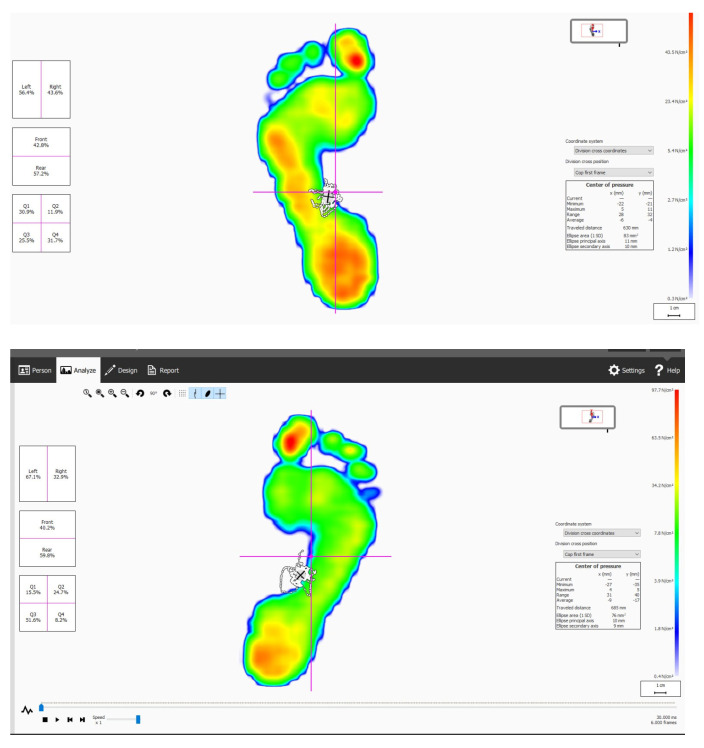
Single-leg balance test measurements displayed on the pedobarographic assessment screen. The image illustrates the center of pressure displacement, including the anterior-posterior and medio-lateral sway parameters.

**Figure 2:**
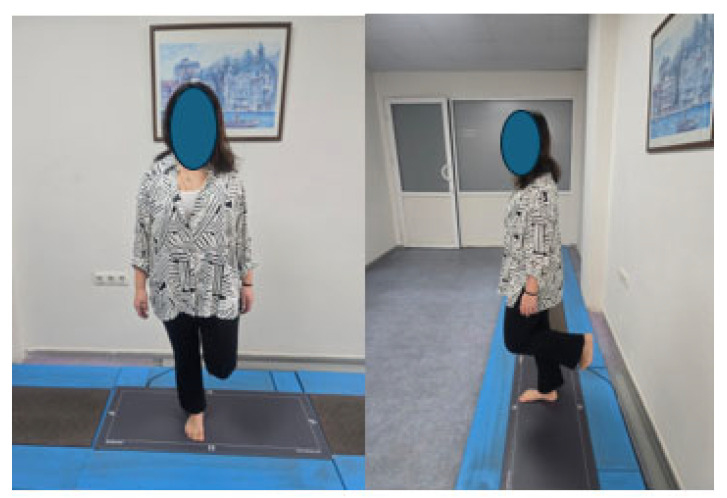
Single-leg balance assessments.

For the balance assessment protocol, the single-leg balance measurements were conducted using a Footscan^®^ pedobarographic measurement system (Rsscan International, Paal, Belgium; 40 × 100 cm, 300 Hz) (Figure 1). The participants were instructed to maintain a single-leg stance on the device with their preferred foot, while the contralateral leg was bent at the knee without contact. The test duration was 30 s, during which participants kept their hands freely by their sides (Figure 2). If balance was lost, the contralateral foot could be placed on the ground and the measurement was immediately terminated. The participants who successfully maintained balance for the full duration had their measurement automatically recorded at the 30th second. Each measurement was repeated three times per foot, and the average values were used for analysis [20].

The test parameters included the following: stance duration, medio-lateral (ML) sway, anterior-posterior (AP) sway and total body sway (TBS), and these were recorded throughout the test.

For the data acquisition and processing, balance assessments were performed under static standing conditions using a Footscan^®^ 9 Scientific pedobarographic system (Materialise Motion, Beringen, Belgium). The system recorded center-of-pressure (CoP) displacement data, providing quantitative measurements for the postural balance analysis.

For the ML sway calculation, ML sway was defined as the standard deviation of the CoP displacement along the mediolateral axis. The CoP data were extracted at a sampling rate of 200 Hz, and the ML sway amplitude was computed using the system’s built-in software.

For the TBS calculations, TBS was assessed by calculating the total trajectory length of the CoP displacement, considering both the AP and ML directions. The CoP displacement was analyzed in millimeters, and the sway patterns were visualized using Footscan’s balance analysis module.

For the data processing, raw CoP signals were processed using a low-pass Butterworth filter with a 10 Hz cutoff frequency, following the manufacturer’s guidelines. The Footscan system automatically generated graphical and numerical outputs, including the CoP trajectory length, force distribution, and pressure maps. All calculations were conducted in accordance with the Footscan technical manual, ensuring standardized data collection and analysis.

Given that limb dominance has been identified as a factor influencing balance in previous studies, both the dominant and non-dominant extremities were analyzed [21]. Limb dominance was determined by asking participants, “Which foot do you use to kick a ball?” or “Which foot would you use to push an object?” [22]. All measurements were performed twice (at baseline (day 0) and at the end of the 8-week follow-up period).

### 2.3. Statistical Analysis

The descriptive statistics of the data are presented as means, standard deviations, medians, minimums, maximums, frequencies, and percentages. The normality assumption for the quantitative data was checked using the Shapiro–Wilk test. For comparisons between the groups regarding the participants’ demographic characteristics, the Independent Samples t-test was used for variables showing normal distribution, while the Mann–Whitney U test was used for variables not meeting the normality assumption. The Pearson Chi-square test was used to examine the relationships between the categorical variables.

When evaluating the changes in balance parameters over time for the control and exercise groups, the non-parametric Brunner–Langer method (F1-LD-F1 design) was used. The analyses were performed using R software version 4.4.1 (R Foundation for Statistical Computing, Vienna, Austria) with the nparLD package. If the Brunner–Langer method indicated that the time-dependent change in groups was not similar (interaction *p* < 0.1), time comparisons were then performed within each group using the Brunner–Langer method (LD-F1 design). Any differences between the groups at each time point after a significant interaction was found were evaluated using the Mann–Whitney U test. IBM SPSS Statistics 25.0 (IBM SPSS Statistics for Windows, Version 25.0. Armonk, NY, USA: IBM Corp.) was used for all statistical analyses. The significance level was set at 0.05 for all analyses (except for interaction, which was *p* < 0.1).

## 3. Results

The mean ages of the participants were 29.7 ± 3.8 years old in the exercise group (EG) and 29.1 ± 6.1 years old in the control group (CG). Regarding lower extremity dominance, 49 participants in both the EG and CG exhibited right foot dominance. The mean BMIs were 25.5 ± 2.2 kg/m^2^ in the EG and 24.9 ± 3.4 kg/m^2^ in the CG. The mean gestational ages were 15.7 ± 3.1 weeks in the EG and 14.8 ± 2.6 weeks in the CG (Table 1).

**Figure 3:**
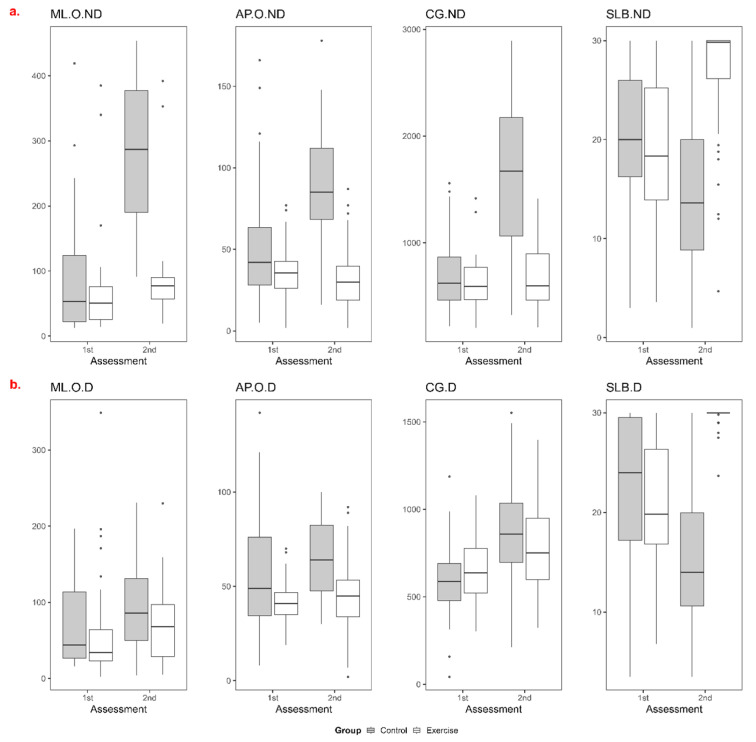
Changes in the balance parameters of the control and exercise groups. ML, mediolateral sway; AP, anterior-posterior sway; CG, center gravity of total body sway; SLB, single-leg balance; O, oscillation; ND, non-dominant; D, dominant. The *y*-axis represents the first and second measurements, while the *x*-axis indicates the values of the measurements. (**a**): non-dominant side; (**b**): dominant side.

The statistical analyses revealed significant group, time, and interaction effects for multiple balance parameters (Table 2). The variations in balance parameters between the exercise and control groups are visually represented as box plots in Figure 3.

The statistical analyses revealed significant group, time, and interaction effects for multiple balance parameters. On the non-dominant side, a minimal increase in medio-lateral (ML) sway was observed in the exercise group (EG), whereas the increase was more pronounced in the control group (CG), with the EG demonstrating a more controlled trajectory (time: *p* = 0.000, group interaction: *p* = 0.000). The within-group analyses indicated that sway could not be maintained in the CG, resulting in a statistically significant increase (group: *p* < 0.0001). On the dominant side, ML sway was initially higher in the CG (group: *p* = 0.047), but by the end of the study, the increase in the EG remained less pronounced (time: *p* = 0.000, group interaction: *p* = 0.512). In terms of anterior-posterior (AP) sway, the EG exhibited a decrease on the non-dominant side, whereas a marked increase was detected in the CG (time: *p* < 0.001, group interaction: *p* = 0.000). The within-group analyses suggested that exercise had a protective effect on this parameter, as the decrease in the EG and the increase in the CG were both statistically significant (group: *p* < 0.0001). On the dominant side, AP sway was higher in the CG at baseline (group: *p* = 0.002) but showed a smaller and more controlled increase in the EG compared to the CG over time (time: *p* < 0.001, group interaction: *p* = 0.002). The within-group analyses indicated increases in both groups; however, the increase in the EG was significantly less pronounced. Regarding total balance sway (TBS), the EG demonstrated a more controlled increase on the non-dominant side (time: *p* = 0.0011, group interaction: *p* = 0.000), whereas the increase in the CG was more pronounced and statistically significant (group: *p* < 0.0001). On the dominant side, although both groups exhibited significant increases, the EG maintained a more controlled trajectory (time: *p* = 0.001, group interaction: *p* = 0.000, group: *p* < 0.0001). In terms of one-leg stance duration (OSD), the EG showed an increase on the non-dominant side despite pregnancy progression (time: *p* < 0.0001, group interaction: *p* = 0.000), whereas a significant decrease was observed in the CG (group: *p* < 0.0001). Similarly, on the dominant side, OSD decreased over time in the CG, while the EG exhibited a statistically significant increase (time: *p* < 0.0000, group interaction: *p* = 0.000), suggesting a positive effect of exercise on balance control.

## 4. Discussion

This study was designed as a retrospective re-evaluation of data from a previously conducted randomized single-blind trial [18]. Since pedobarographic measurements were performed in the reference study, these data were retrospectively retrieved from the system and analyzed for balance-related parameters. The primary objective of this retrospective study was to investigate the effects of clinical exercise interventions during pregnancy on single-leg balance. The findings demonstrated that exercise interventions exert protective and enhancing effects on balance parameters during pregnancy. The risk of falling during walking and other daily activities in pregnancy is a critical concern for both maternal and fetal health. Pregnancy introduces significant biomechanical challenges to bipedal stability, as weight gain and its distribution across different body regions alter the body’s center of mass [23]. Concurrently, sensorimotor system activation adapts to these structural changes, potentially influencing postural control [24]. Notably, falls due to these physiological changes are more frequently reported during the second trimester of pregnancy [25]. Approximately two-thirds of these falls occur while walking on slippery surfaces, hurrying, or carrying an object [8]. Pregnant individuals who develop a fear of falling often reduce their physical activity levels, despite being aware of the benefits of exercise [23]. Regular physical activity and structured exercise programs during pregnancy offer a range of benefits for both maternal and fetal health [26,27]. These include weight-gain control, improved muscle strength, better management of gestational diabetes, and improved psychological well-being [16]. The current study highlights that exercise-based interventions may contribute to balance maintenance, thereby reducing the risk of fall-related complications during pregnancy.

Previous studies have primarily assessed balance during walking and dynamic standing throughout pregnancy, with a focus on detecting longitudinal changes in balance parameters [8,11,23,24]. In the study, significant improvements were observed in medio-lateral (ML) sway, anterior-posterior (AP) sway, total body sway (TBS), and one-leg stance duration (OSD) in the exercise group (EG). Our findings aligned with those of Danna-Dos-Santos et al., who examined balance parameters in pregnant individuals across the first, second, and third trimesters, as well as in non-pregnant controls. Their study demonstrated that both ML and AP sway progressively increased throughout pregnancy [24]. Similarly, McCrory et al. compared the dynamic balance of pregnant individuals with and without a history of falls and healthy non-pregnant controls. Their findings indicated that pregnant individuals who had experienced falls exhibited greater balance impairments, reinforcing the association between pregnancy-related postural adaptations and fall risk [28].

Further supporting our results, El-Shamy et al. investigated AP, ML, and global postural sway in pregnant individuals who underwent a 4-week postural exercise program compared to a non-exercising control group. Consistent with our findings, their study reported improvements in all measured parameters following the intervention in the EG, highlighting the positive impact of structured exercise programs on balance maintenance during pregnancy [25].

This study examined the effects of exercise interventions during pregnancy on balance parameters, with a specific focus on the role of limb dominance in balance performance. The findings revealed that the non-dominant (ND) side exhibited greater changes in medio-lateral (ML) and anterior-posterior (AP) sway. In the exercise group (EG), sway on the ND extremity remained minimal, whereas in the control group (CG), a significant increase was observed. This difference may be attributed to the weaker role of the ND extremity in balance control [14].

Analyses of the dominant (D) extremity revealed increases in sway in both the CG and EG, likely due to pregnancy-induced postural changes. However, the more controlled increase in the EG suggests that exercise may contribute to maintaining balance on the D extremity. In the one-leg stance duration (OSD) analyses, the CG exhibited a significant decline in balance maintenance on the ND side, whereas the EG demonstrated a statistically significant increase in stance duration, despite pregnancy progression. On the D side, both groups exhibited increases in stance duration; however, this improvement was more pronounced in the EG. These findings suggest that exercise enhances single-leg balance by strengthening lower extremity muscles and deep stabilizers [29]. Despite the shifting center of body mass during pregnancy, we hypothesize that the positive effects of exercise on the sensorimotor system contribute to a reduction in sway. Although the influence of limb dominance on balance remains debated, enhanced functionality and centralized postural support on the dominant leg may explain this outcome [14]. Additionally, proprioceptive inputs from joint afferents during exercise may have played a role in improving balance control [25].

## 5. Conclusions

The findings of this study indicate that exercise programs implemented during pregnancy are effective in maintaining and enhancing balance. Notably, exercise plays a critical role in reducing anterior-posterior (AP) sway and increasing one-leg stance duration (OSD), which are key factors in postural stability. These improvements are particularly important in minimizing fall risk and facilitating the safe execution of functional movements throughout pregnancy. Given these benefits, structured clinical exercise interventions should be incorporated into prenatal care programs to enhance balance control and mitigate fall-related risks in pregnant individuals. Implementing such interventions may contribute to maternal well-being and fetal safety by reducing pregnancy-related postural instability.

## Figures and Tables

**Table 1:** Participants’ demographic characteristics.

	Exercise Group (*n* = 50) M ± SD	Control Group (*n* = 51) M ± SD	*p*-Value
Age (years)	29.7 ± 3.8	29.1 ± 6.1	0.505 ^1^
Dominant side Right Left	*n* (%) 49 (98.0) 1 (2.0)	*n* (%) 49 (96.0) 2 (4.0)	0.063 ^2^
BMI (kg/m^2^)	24.5 ± 2.2	24.9 ± 3.4	0.491 ^1^
Height (cm)	164.0 ± 6.8	161.7 ± 6.7	0.084 ^1^
Body weight (kg)	66.0 ± 8.4	65.0 ± 9.8	0.584 ^1^
Gestational Week	15.7 ± 3.1	14.8 ± 2.6	0.166 ^2^

SD, standard deviation; min.–max., minimum-maximum; cm, centimeter; n, number of people; kg, kilogram; BMI, body mass index; BW, body weight; ^1^, independent sample *t*-test; ^2^, Mann–Whitney U test.

**Table 2:** Changes in Postural Sway and Balance Parameters Over Time in the Experimental and Control Groups.

Measurement	EG	EG	CG	CG	*p*-Value	*p*-Value	*p*-Value
	(Baseline)	(Week 8)	(Baseline)	(Week 8)	(Group)	(Time)	(Interaction)
ML sway							
ND	51 (14–385)	77 (19–392)	53 (13–419)	287 (91–454)	0.000	0.000	0.000
D	34 (2–349)	68 (5–230)	44 (16–197)	86 (4–231)	0.047	0.000	0.512
AP sway							
ND	35.5 (2–77)	30 (2–87)	42 (5–166)	85 (16–178)	0.000	0.005	0.000
D	41 (19–70)	45 (2–92)	49 (8–142)	64 (30–100)	0.000	0.000	0.002
TBS							
ND	592 (205–1416)	598.5 (209–1416)	620 (218–1558)	1672 (324–2894)	0.000	0.011	0.000
D	637.5 (303–1080)	752 (322–1397)	587 (42–1187)	858 (213–1552)	0.000	0.000	0.000
OSD second							
ND	18.35 (3.6–30)	29.85 (4.7–30)	20 (3–30)	13.6 (1–30)	0.000	0.000	0.000
D	19.81 (6.8–30)	30 (23.7–30)	24 (3.5–30)	14 (3.5–30)	0.000	0.000	0.000

ML, mediolateral; AP, anterior-posterior; TBS, total body sway; OSD, one-leg stance duration; EG, exercise group; CG, control group.

## Data Availability

The data presented in this study are available upon request from the corresponding author due to the principles of the Personal Data Protection Law in Turkey.

## Supplementary Materials

The following supporting information can be downloaded at: https://www.mdpi.com/article/10.3390/jcm14061892/s1, File S1. Exercise Training Protocol.

## Abbreviations

The following abbreviations are used in this manuscript:

ML	Mediolateral
AP	Anterior-posterior
TBS	Total body sway
OSD	One-leg stance duration
