# Supplementary material for: Retrospective Analysis of Balance Parameters in Pregnant Women: A Sub-Analysis of a Randomized Controlled Trial
Source: J Clin Med. 2025 Mar 11;14(6):1892. doi: 10.3390/jcm14061892 (PMC11943415; doi:10.3390/jcm14061892)
Supplement: Supplementary file 1 [file jcm-14-01892-s001.zip › jcm-3490684-supplementary.pdf]

Exercise Training Protocol

The clinical exercise training session is structured into warm-up, loading, and cool-down phases.

In the warm-up phase of the training:

Deep diaphragmatic breathing exercises, light-paced walking, and postural awareness exercises were conducted while standing, seated on a large exercise ball, or sitting on a chair. Postural awareness exercises emphasized maintaining the pelvis in a neutral position, ensuring the distance between the two medial malleoli, full foot contact with equal weight distribution on the toes and heels, and a relaxed position of the hips, knees, and ankles. The exercises also included maintaining the neutral curvature of the spine, aligning the thorax to a neutral position, achieving the plane between the last rib and the pelvic bone, and ensuring the upper extremities and neck were in a neutral position.

Deep diaphragmatic breathing (DDB) involved the slow and full contraction of the diaphragm during inhalation, allowing abdominal expansion, and the slow and complete contraction of abdominal muscles during exhalation, resulting in abdominal compression (Liu et al., 2023). The duration of the warm-up phase ranged from 5 to 10 minutes, depending on the participant's needs.

In the loading phase of the training:

The loading phase utilized large exercise balls, small exercise balls, free weights, elastic bands, or body weight. All exercises during this phase were performed with a neutral spine and body awareness in seated, standing, and crawling positions. Resistance training with elastic bands was adjusted according to the perceived exertion scale (RPE) following the recommendations of the American College of Sports Medicine (ACSM). Loading exercises were conducted at a moderate intensity, corresponding to a score of 4–5 on the Modified Borg Scale.

Initially, exercises typically began with 6–8 repetitions and progressed to 8–10 repetitions. The number of sets started at 1–2 and advanced to 2–3. Intensity was reassessed weekly and increased accordingly. Repetition count was first increased, followed by the number of sets in subsequent sessions based on participant feedback (Page P., Ellenbecker T., 2021).

elvic mobility exercises included movements such as anterior-posterior pelvic tilts and asymmetrical pelvic motions. Pelvic floor muscle exercises were implemented with a focus on strengthening and maintaining endurance until the 28th week of pregnancy (Soave et al., 2019).

Shoulder and upper extremity mobility exercises were performed using proprioceptive neuromuscular facilitation (PNF) patterns. (M. M. Ribeiro et al., 2021).

Specific spinal exercises target the local muscles of the lumbopelvic region. These exercises focus on the local muscle system, which includes deep muscles such as the transversus abdominis and lumbar multifidus that attach directly to the lumbar vertebrae and sacrum, enabling precise control of lumbar segments.

In contrast, the global muscle system involves larger, more superficial trunk muscles, such as the external obliques and erector spinae, which are primarily responsible for producing and controlling trunk movements. These exercises aim to facilitate the co-contraction of the transversus abdominis and lumbar multifidus independently of other major trunk muscles (Richardson et al., 2002).

Strengthening exercises for the upper extremity and scapular muscles, as well as for the lower extremity muscles, were integrated with breathing techniques during training. Free weights, resistance bands, or body weight were utilized in the strengthening exercises (Barakat, 2021). The loading phase lasted approximately 30 minutes.

In the cooling-down phase of the training:

Relaxation and stretching exercises were conducted during the cooling-down phase. These exercises were performed in comfortable positions on a mat or while seated on a chair, targeting major muscle groups. This phase lasted approximately 10 minutes.

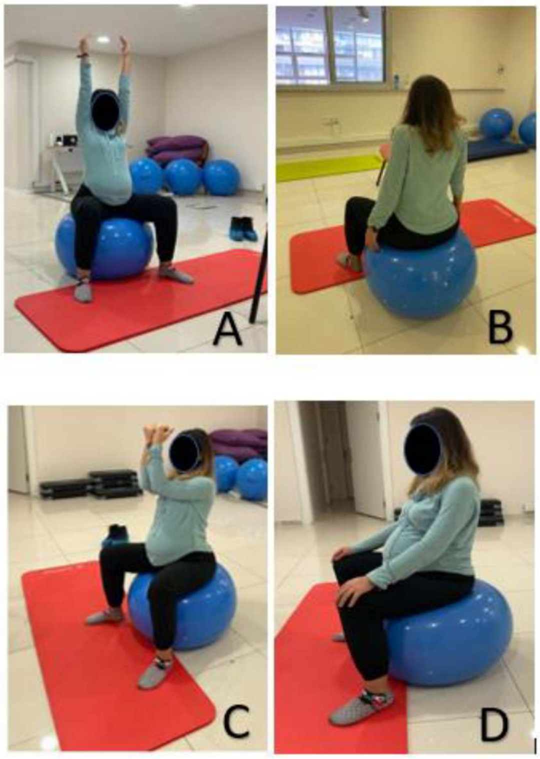

Exercise Training Examples with a Large Exercise Ball (A: Shoulder Mobility Exercise; B: Shoulder Elevation Exercise; C: Scapular Abduction Exercise; D: Pelvic Floor Exercises)

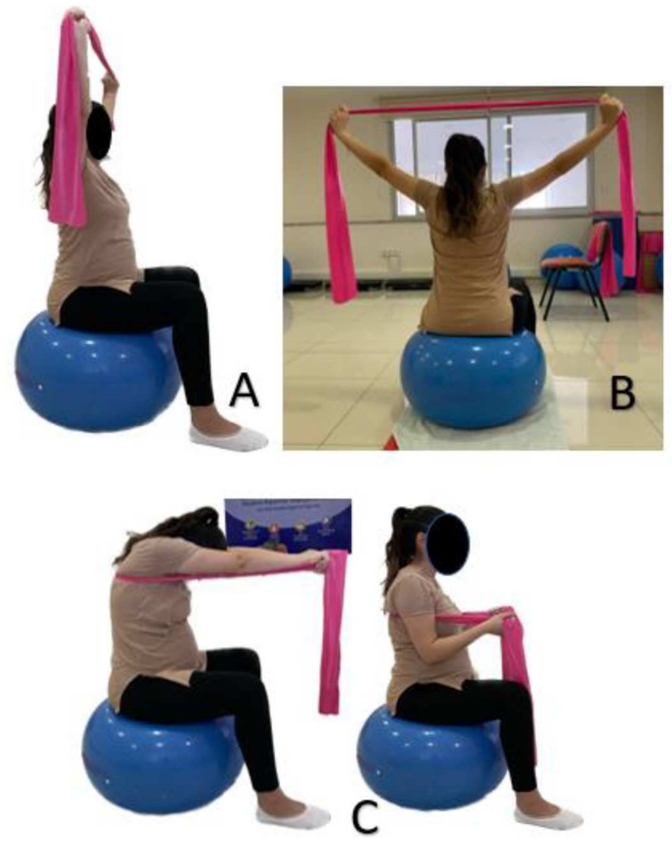

Upper Extremity Strengthening Training Examples on a Large Exercise Ball (A: Shoulder Flexion Strengthening; B: Shoulder Adduction in Flexion Exercise; C: Serratus Anterior Strengthening + Thoracic-Cervical Mobility)
